# Development of Simple Sequence Repeat (SSR) Markers from a Genome Survey of a *Cymbidium kanran* Makino Population in Jeju Island, Republic of Korea

**DOI:** 10.4014/jmb.2501.01013

**Published:** 2025-02-24

**Authors:** Kyeoung Cheol Kim, Seungtae Kang, Su-Lim Kim, Rambukkana Maggonage Thiruni Dananjana Perera, Jin Kyu Woo, Kumarasinghe Hiruni Sandunika, Ji-Hyang Kim, Dong-Sun Lee

**Affiliations:** 1 Bio-Health Materials Core-Facility Center, Jeju National University 63243, Jeju, Republic of Korea; 2 Department of heritage policy, world heritage office, Jeju special self-governing province 63122, Republic of Korea; 3 Interdisciplinary Graduate Program in Advanced Convergence Technology and Science, Jeju National University, Jeju 63243, Republic of Korea; 4 Xenohelix Research Institute, Incheon 21984, Republic of Korea; 5 Subtropical/Tropical Organism Gene Bank, Jeju National University 63243, Jeju, Republic of Korea; 6 Faculty of Biotechnology, College of Applied Life Sciences, Jeju National University, Jeju 63243, Republic of Korea

**Keywords:** SSR, *Cymbidium*, conservation, genetic diversity

## Abstract

The *Cymbidium kanran* Makino, an economically significant ornamental plant, is observed in small numbers in its natural habitat on Jeju Island in South Korea. *C. kanran* of Jeju is afforded protection due to a decline in its population resulting from environmental changes and illegal poaching. We developed simple sequence repeat (SSR) markers to analyze the differences to other *C. kanran* through molecular genetic studies. Based on the results of the Random amplified polymorphic DNA (RAPD) analysis and whole genome sequencing, 86 initial SSR marker candidates were selected *in silico*. After excluding those that were structurally unsuitable, 40 were reselected through polymorphism testing. Finally, 25 markers were selected based on the diversity test and their applicability to real samples. The newly developed markers will prove invaluable in substantiating the distinctiveness of *C. kanran* from Jeju, as well as in the processes of conservation, restoration, and the identification of cultivars.

## Introduction

*Cymbidium* is an important horticultural plant genus with high economic and ornamental value [1]. Therefore, characterizing these species is vital for the management, conservation, and understanding of their genetic relationships [2]. In the Republic of Korea, 92 species, 11 varieties, and 7 cultivars of Orchidaceae plants grow naturally. Among these, Cymbidium species such as *Cymbidium kanran*, *C. koran*, *C. lancifolium*, *C. javanicum* var. as pidistrifolium, *C. nipponicum*, and *C. goeringii* are distributed on the southern slopes of Hallasan Mountain on Jeju Island. *C. kanran* was first described in 1900 by the Japanese botanist Kino. It is found in temperate southern climates across several regions, including Jeju Island in the Republic of Korea, Southern Japan, Taiwan, southern China, and Yunnan Province. The natural habitat of *C. kanran* on Jeju Island has been destroyed by the development of pastures and citrus orchards, and the species faces extinction owing to illegal poaching [3].

The Jeju Sanghyo-dong *C. kanran* habitat (natural monument no. 432) is the only location in the Republic of Korea where the flowering of *C. kanran* has been observed [4]. Therefore, it is necessary to adopt immediate measures to conserve these species. Before developing conservation measures for any species, it is crucial to understand its genetic diversity and organization, which requires effective marker resources. In the past, *Cymbidium* biodiversity has been evaluated based on morphological and physiological traits; however, this approach has constraints because environmental factors influence these characteristics [2].

Incorporating the ploidy, chromosome number, and reproductive system of *C. kanran* would indeed enhance the understanding of its biological characteristics. *C. kanran* generally exhibits a diploid chromosome number of 40. Natural self-pollination (autogamy) is absent in this species, meaning it requires external pollination to produce seeds. However, the natural fruit set rate is significantly lower than that achieved through artificial pollination, indicating a substantial restriction in natural pollination. This species primarily relies on insect pollination for reproduction. The unique floral features of *C. kanran*, including the specialized labellum, pollinia, and gynandrium, evolved through complex interactions with pollinators. While many orchids use nectar to attract pollinators, about one-third of species, including *C. kanran*, employ deceptive strategies such as sexual and food deception. A study by Japanese researchers demonstrated that the unique floral features of *C. kanran* including its specialized labellum, pollinia, and gynandrium evolved through complex interactions with pollinators. The species produces specific volatile compounds to attract Apis cerana japonica for pollination, despite not offering a food reward [5, 6].

Molecular markers are increasingly used to understand population genetic structure, parentage, population viability, gene flow, and genetic diversity, as well as to study synthetic pathways, evolution, the effects of habitat fragmentation, and to guide conservation strategies [7, 8]. Molecular markers used to analyze genetic polymorphisms are categorized into protein- and DNA-based markers. While early markers such as allozymes were simple and neutral, they were replaced by DNA-based markers owing to low polymorphism and environmental variation. Among DNA markers, restriction fragment length polymorphism (RFLP) and variable number tandem repeat (VNTR) have advantages such as high transferability but are costly and labor intensive. PCR-based markers, such as random amplified polymorphic DNA (RAPD), simple sequence repeat (SSR), and single nucleotide polymorphisms (SNPs), are more commonly used. RAPD does not require sequence data but is a dominant marker, whereas SSR and SNPs require sequence data, are co-dominant, and show high polymorphisms [9, 10]. Among PCR-based markers, Simple Sequence Repeat (SSR) markers have become widely adopted due to their high polymorphism, codominant inheritance, and extensive applicability in genetic studies. SSRs are short, tandemly repeated DNA sequences distributed throughout the genome. Their high variability, stemming from differences in repeat numbers, makes them invaluable tools for assessing genetic diversity, population structure, and gene flow. The codominant nature of SSRs allows for the detection of both alleles in heterozygous individuals, offering more comprehensive genetic insights compared to dominant markers such as RAPD. Additionally, SSR markers are highly reproducible, capable of detecting even subtle genetic differences, and are found across a wide range of species.

This study focused on developing SSR markers specific to *C. kanran* as a crucial step toward understanding its genetic diversity and population dynamics, providing powerful tools to support conservation efforts and secure the long-term survival of this endangered orchid species.

## Materials and Methods

### *C. kanran* Samples

A total of 20 *C. kanran* leaf samples were obtained from Jeju Special Self-Governing Province World Heritage Headquarters. Details are provided in the following Table 6 and Fig. S1. Genomic DNA (gDNA) was extracted from the provided samples using the Bio-medic Plant gDNA Extraction Kit (www.ibiomedic.co.kr). The extracted gDNA was quantified using a DS-11 Spectrophotometer (DeNovixDS, USA, in Bio-Health Materials Core-Facility, Jeju National University) and verified by 1% (w/v) agarose gel electrophoresis.

### Genomic DNA Extraction

As part of the preparatory process for whole-genome sequencing (WGS) of *C. kanran* reference samples, RAPD PCR was performed using gDNA extracted from 16 provided samples as templates. Allelic data obtained from RAPD PCR were used to construct UPGMA and Neighbor-Joining (NJ) dendrograms for genetic relationship analysis.

### RAPD Analysis

Whole-genome sequencing was performed on two *C. kanran* samples (JCK-01, JCK-07). gDNA was extracted from both samples and paired-end whole-genome sequencing was conducted using the Illumina NovaSeq6000 platform. Given the estimated genome size of *Cymbidium* species, approximately 60 Gb of raw sequencing data were generated for each sample. Libraries for whole-genome sequencing were prepared using the Illumina TruSeq Nano DNA Library Kit. The UPGMA phylogenetic tree was constructed using MEGA software (version 7.0; [11]).

### Whole Genome Sequencing of *C. kanran* Samples

PCR was performed using HSTM Taq PCR polymerase (Dongsheng Biotech, China) and ABI 2720 thermal cycler (Applied Biosystems, USA). PCR reaction condition and amplication condition are shown following Table 7.

### DNA Fragment Analysis

PCR amplification products was diluted to 1/50 using D.W. Diluted sample 1 ul was added to 9 ul Hi-Di Formamide (Applied biosustems): GeneScan 500 LIZ size standard (Applied Biosystems) (99:1) mixture and it was denaturated at 95°C for 2 min. After cooling down on ice, Fragment analysis was performed with 3730 DNA Analyzer equipped with 50 cm capillary. DNA fragment size was analyzed using GeneMapper software (ver.4.0; Applied Biosystem). Genetic parameters, including major allele frequency (MAF), number of alleles (NA), genetic diversity (GD), and polymorphism information content (PIC), were measured by calculating shared allele frequencies using PowerMarker software (version 3.25; [12]).

## Results

### Identification of SSR Candidate Markers through Whole-Genome Sequencing and Comparative Genomic Analysis

**Fig. 1:**
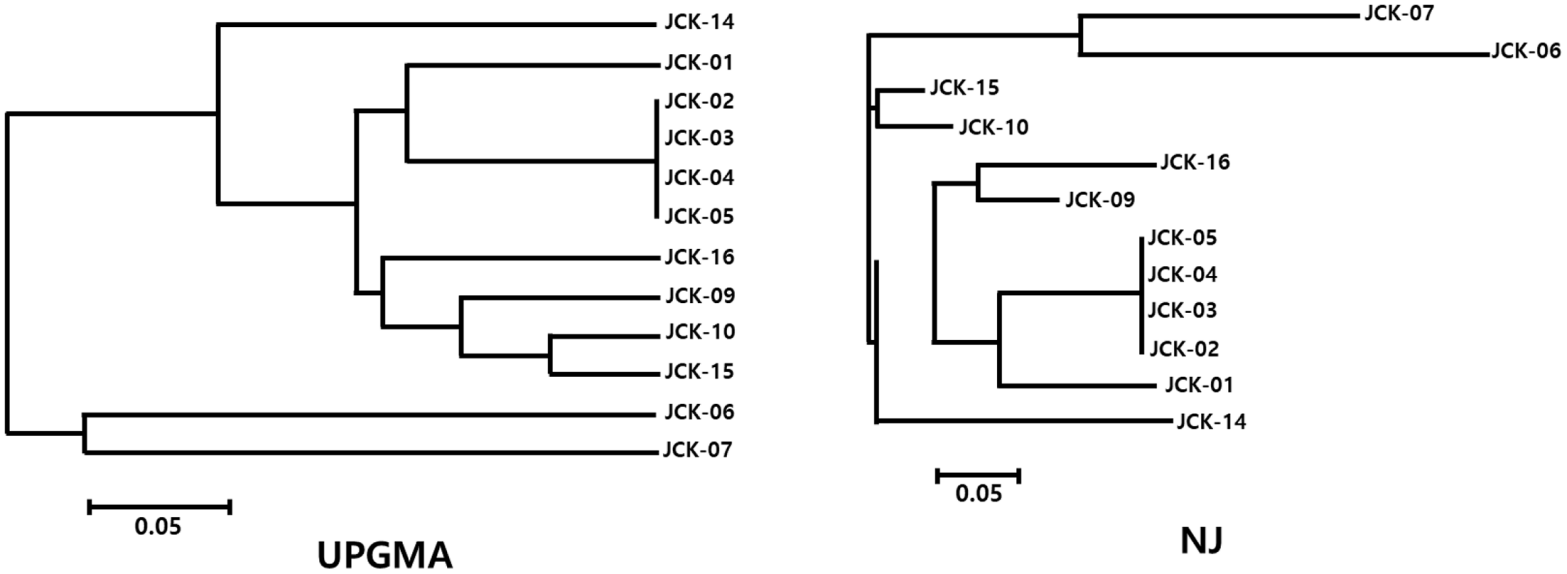
UPGMA and Neighbor-Joining (NJ) dendrograms based on allele information obtained from RAPD PCR. The tree is unrooted due to the absence of an outgroup and represents genetic relationships among *C. kanran* samples.

To perform whole-genome sequencing (WGS), RAPD PCR (Random Amplified Poly-morphic DNA Polymerase Chain Reaction) was conducted on 16 *C. kanran* samples. Genetic relationships among the samples were assessed by constructing dendrograms using the Neighbor-Joining (NJ) method (Fig. 1). The dendrogram revealed that four native habitat samples (JCK-02, JCK-03, JCK-04, and JCK-05) were genetically identical or highly similar, indicating low genetic variation among them. In contrast, JCK-01 and JCK-07 exhibited greater genetic divergence and were therefore selected for whole-genome sequencing.

A review of available genome data for the genus *Cymbidium* showed that no genomic information was available for *C. kanran*, while the average genome size of four closely related species was approximately 4 Gb (Table 1). WGS was designed to achieve 15x genome coverage for *C. kanran*. Paired-end sequencing generated 73.8 Gb and 75.4 Gb of raw data for JCK-01 and JCK-07, respectively, yielding approximately 500 million reads for JCK-01 and 490 million reads for JCK-07. The GC content was 33.17% and 32.99% for JCK-01 and JCK-07, respectively (Table S1).

The raw sequencing data were preprocessed and trimmed, followed by de novo genome assembly to construct contigs. Microsatellite-containing contigs were identified, and multiple sequence alignment was performed using the CLC Genomics Workbench. As a result, 86 candidates for polymorphic SSR markers were identified through *in silico* analysis.

### Polymorphism of Identified SSR Candidate Markers

**Fig. 2:**
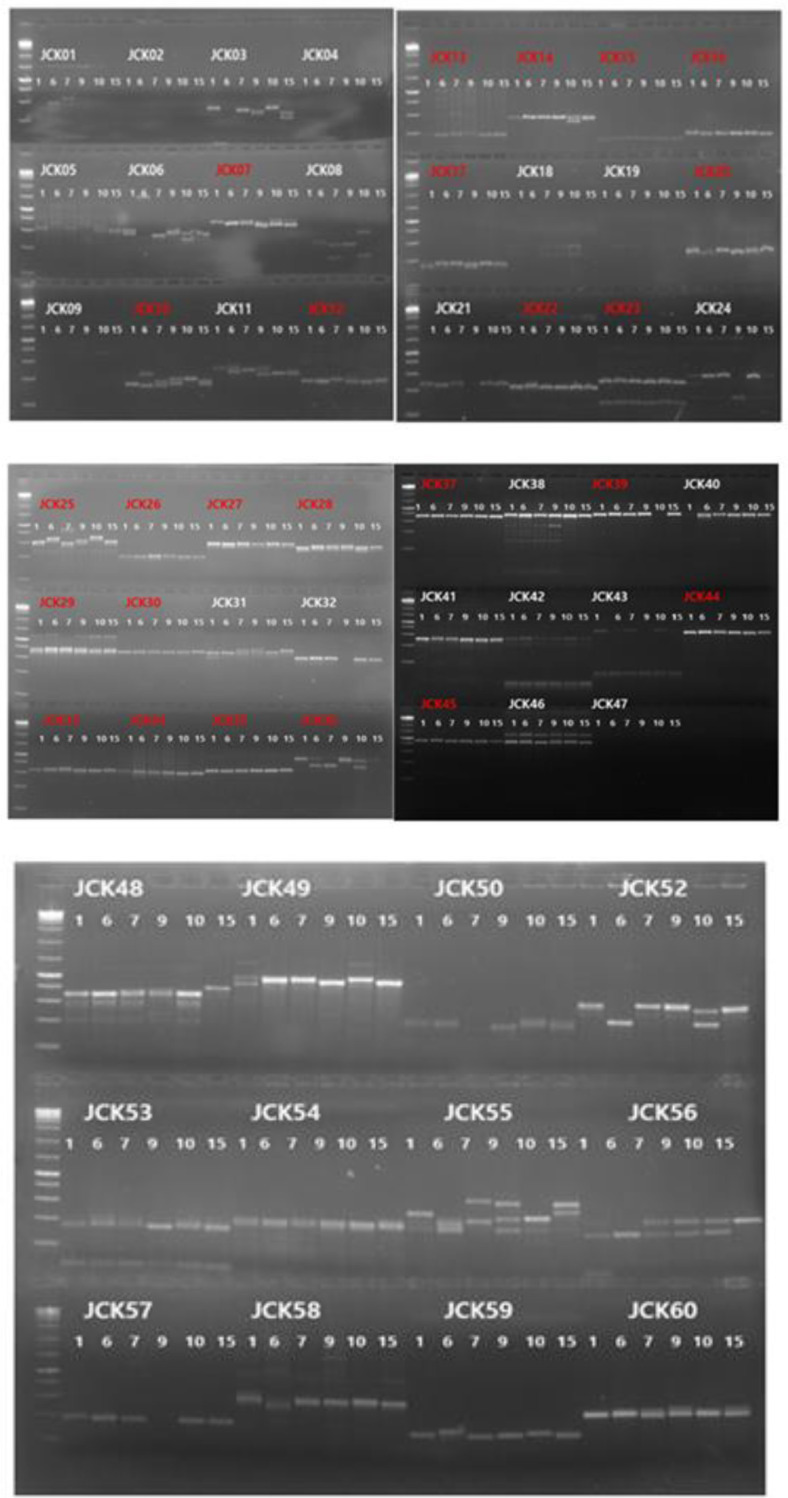
Polymorphism testing of 59 SSR marker candidates using six *C. kanran* samples. PCR amplification products were electrophoresed on a 3% (w/v) agarose gel in 0.5x TAE running buffer. Markers highlighted in red were selected for fragment analysis using FAM-modified primers.

Among the 86 identified polymorphic SSR candidate markers, 59 primers were designed after excluding those prone to hairpin, self-dimer, or hetero-dimer formation. A complete list of the synthesized primers is provided in Table 2. Six *C. kanran* samples (JCK-01, JCK-06, JCK-07, JCK-09, JCK-10, and JCK-15) were analyzed using the 59 SSR markers. After applying selection criteria based on polymorphism, PCR amplification clarity, and efficiency, 40 markers were retained (Fig. 2 and Table 3).

### Genetic Diversity Analysis Using Discovered SSR Markers

**Fig. 3:**
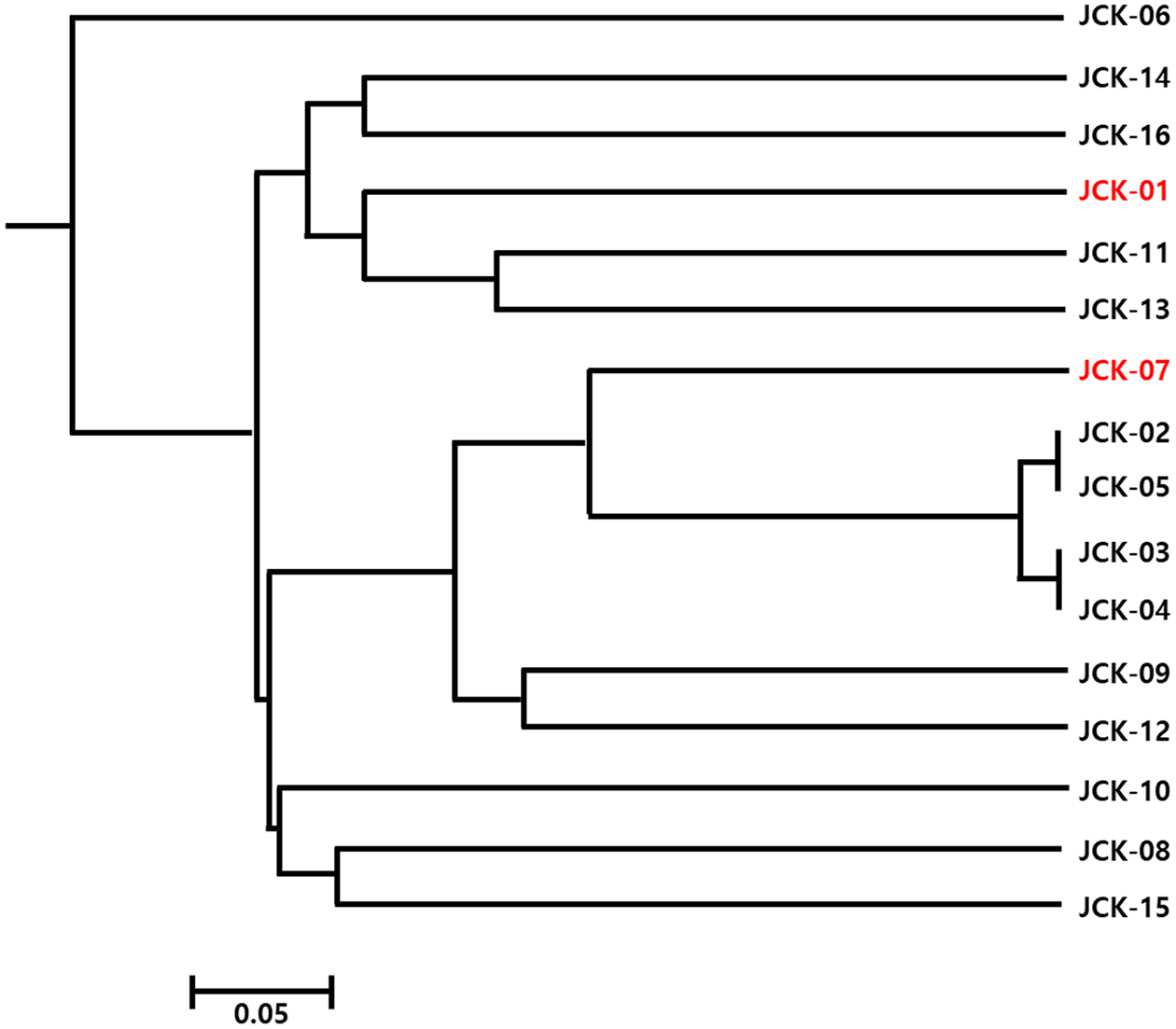
UPGMA dendrogram constructed using 40 SSR markers applied to 16 *C. kanran* samples. The tree is unrooted, reflecting genetic clustering without an evolutionary reference point.

Genetic diversity analysis of the initial 16 *C. kanran* samples, using 40 SSR markers, revealed substantial genetic variation among most samples. The UPGMA dendrogram (Fig. 3) indicated clear genetic separation; however, some samples displayed identical allele patterns. Specifically, JCK-02, JCK-03, JCK-04, and JCK-05 shared identical alleles across all 40 loci, suggesting high genetic similarity or possible redundancy among these individuals.

A detailed summary of the genetic characteristics of the 40 SSR loci is provided in Table 4. This table includes key metrics such as Major Allele Frequency (MAF), representing the frequency of the most common allele at each locus; Number of Alleles (NA), indicating observed allelic diversity; and Polymorphism Information Content (PIC), reflecting the informativeness of each marker for genetic diversity assessment.

The genetic variability at each SSR locus was quantified based on allele count, heterozygosity, gene diversity, and PIC. An average of 6.2 alleles per locus was detected among the 246 identified alleles. The MAF ranged from 0.16 to 0.97, while the number of detectable alleles (NA) per SSR marker varied between 2 and 15. The PIC values ranged from 0.06 to 0.90, indicating a wide range of polymorphism levels across the loci. These results demonstrate the potential of the selected SSR markers for assessing genetic diversity in *C. kanran*.

### Final Selection of High-Quality SSR Markers and Genetic Diversity Analysis

Following a comprehensive evaluation of the 40 initially selected SSR markers, a final subset of 25 high quality markers was chosen based on key selection criteria. These criteria included functionality across multiple samples, detection of two or fewer alleles per plant, and the absence of amplification artifacts (Table 3; markers indicated in red).

**Fig. 4:**
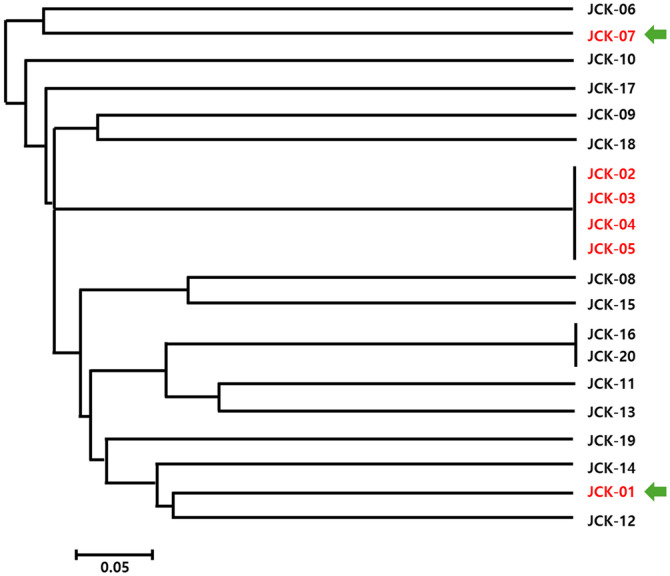
UPGMA dendrogram constructed using 25 SSR markers applied to 20 *C. kanran* samples. Green arrowheads indicate the samples used for whole-genome sequencing. The tree is unrooted due to the lack of an outgroup, showing genetic relationships among the samples.

To enhance the reliability of the genetic diversity analysis, four additional samples were included, resulting in a total of 20 *C. kanran* samples being analyzed using the 25 selected SSR markers. A new UPGMA dendrogram was constructed based on allele-sharing patterns among the samples (Fig. 4), revealing broader genetic differentiation. This updated dendrogram provided more comprehensive insights into the genetic diversity and population structure of *C. kanran*.

The genetic characteristics of the 25 SSR loci applied to the 20 samples are summarized in Table 5. Key metrics, including allele frequency, number of alleles per locus, and Polymorphism Information Content (PIC), were evaluated, confirming the effectiveness of the selected markers in detecting genetic variation. These results further support the applicability of the identified markers for future population genetics and conservation studies of *C. kanran*.

## Discussion

This study aimed to explore the genetic diversity of *C. kanran* and develop polymorphic SSR markers through whole-genome sequencing and comparative genomic analysis [8, 17]. As *C. kanran* is currently classified as an endangered species in Korea, the development of a specific marker for *C. kanran* native to Jeju Island is imperative. The findings provide important insights into the genetic structure, marker development, and potential applications for conservation and breeding programs. Despite the research conducted on *C. kanran*'s markers, these studies have employed outdated technology, and the extant literature is rather limited. The first reported marker was the ERAPD marker, so its specificity and reproducibility were lower than those of currently used common markers, and most of the other markers were studied using Chinese native species [18-20]. The development of marker candidates for the genetic analysis of individuals adapted to Jeju environmental conditions was based on the most recent technologies, such as NGS and *in silico* methods. As a result, fourteen novel SSR markers were previously identified in *Cymbidium* spp. and successfully employed to measure the genetic diversity and relationships within a *Cymbidium* collection [1]. Moreover, the fourteen identified SSR markers were successfully applied in a genetic diversity study of *Cymbidium* species in the Republic of Korea, revealing that *C. goeringii* is more abundant and widespread in Korea compared to *C. sinensis* [21, 22].

The initial RAPD PCR analysis and dendrogram construction revealed that four native habitat samples (JCK-02, JCK-03, JCK-04, and JCK-05) were genetically identical or highly similar, indicating limited genetic variation among these individuals. This lack of diversity may result from habitat fragmentation, clonal propagation, or restricted gene flow. Similar patterns have been reported in other orchid species under environmental stress or isolated conditions. These findings highlight the necessity of conservation strategies aimed at preserving or increasing genetic diversity within native populations. Whole-genome sequencing of two genetically distinct samples, JCK-01 and JCK-07, produced high-quality genomic data with approximately 15x genome coverage, confirming the feasibility of genome-wide SSR marker discovery. The GC content (≤ 33%) was consistent with values reported in related orchid species, validating the sequencing results. A total of 86 candidate polymorphic SSR markers were identified through *in silico* analysis, demonstrating the utility of high-throughput sequencing in marker development for non-model plant species. A systematic screening process resulted in the selection of 40 polymorphic SSR markers after removing loci with low amplification efficiency or limited polymorphism. These markers were tested on 16 samples, revealing an average of 6.2 alleles per locus, with PIC values ranging from 0.06 to 0.90 [23]. The observed allele diversity confirmed the effectiveness of these markers in capturing genetic variation [24, 25]. Further evaluation led to the final selection of 25 high-quality SSR markers based on key criteria such as consistent amplification, the detection of two or fewer alleles per plant, and the absence of amplification artifacts. To enhance the reliability of the genetic diversity analysis, four additional samples were included, bringing the total to 20 analyzed samples. A new UPGMA dendrogram constructed using these samples provided a clearer view of the population structure and genetic relationships. Broader genetic differentiation was observed, suggesting a more comprehensive representation of *C. kanran*'s genetic diversity. The present study was conducted with a very limited number of 20 samples due to the restrictions imposed by the Korean collecting regulations. This was sufficient for our goal of identifying *C. kanran* of Jeju Island with higher accuracy, rather than the entire *C. kanran* species. However, the utilization of the established markers for the comprehensive identification of *C. kanran* species may prove to be a more efficacious approach than the markers that are currently presented.

The identification of genetically similar individuals within native habitats underscores the urgent need for conservation measures to preserve genetic diversity. Strategies such as habitat restoration, gene flow enhancement, and introducing genetically diverse individuals could strengthen population resilience. Additionally, the developed markers have several applications, including cultivation identity/assessment of purity, assessment of genetic diversity and parental selection, identification of genomic regions under selection, and marker-assisted backcrossing [26-28]. This novel set of SSR markers makes the breeding process more precise and efficient, ultimately contributing to the preservation, breeding and genetic monitoring of *C. kanran*. Overall, this study successfully developed and validated a set of robust SSR markers for *C. kanran*, providing a foundation for future research in population genetics, conservation, and breeding. The combination of genome-wide sequencing, marker selection, and expanded genetic analysis highlights the power of molecular tools in advancing conservation genomics for rare and ecologically significant plant species.

## Supplemental Materials

Supplementary data for this paper are available on-line only at http://jmb.or.kr.



## Figures and Tables

**Table 1:** Genome size information of plants in the genus Cymbidium.

Genus	Species	Subspecies	DNA amount 1C (Mbp)	Original reference
*Cymbidium*	*sinense*		3136.00	Jones *et al*., 1998 [13]
*Cymbidium*	*ceres*		4018.00	Capesius, 1976 [14]
*Cymbidium*	*pumilum*	cv. Gareth Latangor	4312.00	Nagl and Capesius, 1977 [15]
*Cymbidium*	*pendulum*		4606.00	Narayan *et al*., 1989 [16]

**Table 2:** Polymorphic SSR marker candidates from *in silico* comparative genomic analysis.

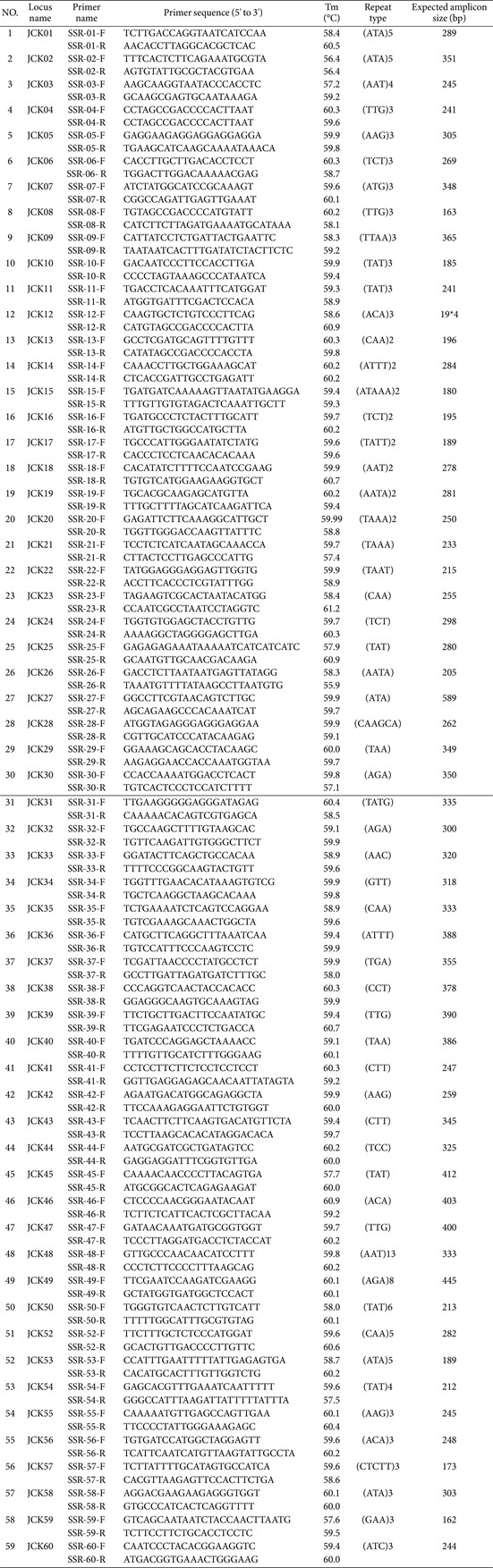

**Table 3:** List of FAM-modified forward primers.

Primer name	Primer sequence (5' to 3')	Repeat type	Expected amplicon size (bp)
FAM(03)-JCK-F	AAGCAAGGTAATACCCACCTC	(ATG)3	348
FAM(06)-JCK-F	CACCTTGCTTGACACCTCCT	(AAT)4	245
FAM(07)-JCK-F	ATCTATGGCATCCGCAAAGT	(TCT)3	269
FAM(10)-JCK-F	GACAATCCCTTCCACCTTGA	(TAT)3	185
FAM(11)-JCK-F	TGACCTCACAAATTTCATGGAT	(TAT)3	241
FAM(12)-JCK-F	CAAGTGCTCTGTCCCTTCAG	(ACA)3	194
FAM(13)-JCK-F	GCCTCGATGCAGTTTTGTTT	(CAA2	196
FAM(14)-JCK-F	CAAACCTTGCTGGAAAGCAT	(ATTT)2	284
FAM(15)-JCK-F	TGATGATCAAAAAGTTAATATGAAGGA	(ATAAA)2	180
FAM(16)-JCK-F	TGATGCCCTCTACTTTGCATT	(TCT)2	195
FAM(20)-JCK-F	GAGATTCTTCAAAGGCATTGCT	(TAAA)2	250
FAM(22)-JCK-F	TATGGAGGGAGGAGTTGGTG	(TAAT)	215
FAM(23)-JCK-F	TAGAAGTCGCACTAATACATGG	(CAA)	255
FAM(26)-JCK-F	GACCTCTTAATAATGAGTTATAGG	(AATA)	205
FAM(27)-JCK-F	GGCCTTCGTAACAGTCTTGC	(ATA)	289
FAM(28)-JCK-F	ATGGTAGAGGGAGGGAGGAA	(CAAGCA)	262
FAM(30)-JCK-F	CCACCAAAATGGACCTCACT	(AGA)	350
FAM(31)-JCK-F	TTGAAGGGGGAGGGATAGAG	(TATG)	335
FAM(32)-JCK-F	TGCCAAGCTTTTGTAAGCAC	(AGA)	300
FAM(33)-JCK-F	GGATACTTCAGCTGCCACAA	(AAC)	320
FAM(34)-JCK-F	TGGTTTGAACACATAAAGTGTCG	(GTT)	318
FAM(35)-JCK-F	TCTGAAAATCTCAGTCCAGGAA	(CAA)	333
FAM(36)-JCK-F	CATGCTTCAGGCTTTAAATCAA	(ATTT)	388
FAM(37)-JCK-F	TCGATTAACCCCTATGCCTCT	(TGA)	355
FAM(38)-JCK-F	CCCAGGTCAACTACCACACC	(CCT)	378
FAM(39)-JCK-F	TTCTGCTTGACTTCCAATATGC	(TTG)	390
FAM(40)-JCK-F	TGATCCCAGGAGCTAAAACC	(TAA)	386
FAM(41)-JCK-F	CCTCCTTCTTCTCCTCCTCCT	(CTT)	247
FAM(42)-JCK-F	AGAATGACATGGCAGAGGCTA	(AAG)	259
FAM(44)-JCK-F	AATGCGATCGCTGATAGTCC	(TCC)	325
FAM(45)-JCK-F	CAAAACAACCCCTTACAGTGA	(TAT)	412
FAM(46)-JCK-F	CTCCCCAACGGGAATACAAT	(ACA)	403
FAM(48)-JCK-F	GTTGCCCAACAACATCCTTT	(AAT)13	333
FAM(49)-JCK-F	TTCGAATCCAAGATCGAAGG	(AGA)8	445
FAM(50)-JCK-F	TGGGTGTCAACTCTTGTCATT	(TAT)6	213
FAM(52)-JCK-F	TTCTTTGCTCTCCCATGGAT	(CAA)5	282
FAM(53)-JCK-F	CCATTTGAATTTTTATTGAGAGTGA	(ATA)5	189
FAM(57)-JCK-F	TCTTATTTTGCATAGTGCCATCA	(CTCTT)3	173
FAM(59)-JCK-F	GTCAGCAATAATCTACCAACTTAATG	(GAA)3	162
FAM(60)-JCK-F	CAATCCCTACACGGAAGGTC	(ATC)3	24

**Table 4:** Genetic characteristics of 40 SSR markers analyzed in 16 *C. kanran* samples.

Marker	SS	NOBS	Availability	NG	MAF	NA	GD	Het	PIC
(03)SSR	16	16.0	1.00	7.0	0.38	9.0	0.79	0.31	0.77
(06)SSR	16	16.0	1.00	11.0	0.22	14.0	0.88	1.00	0.87
(07)SSR	16	16.0	1.00	9.0	0.44	7.0	0.71	0.81	0.67
(10)SSR	16	16.0	1.00	12.0	0.34	11.0	0.83	0.81	0.81
(11)SSR	16	16.0	1.00	10.0	0.16	15.0	0.90	0.69	0.90
(12)SSR	16	15.0	0.94	8.0	0.43	7.0	0.73	0.60	0.69
(13)SSR	16	15.0	0.94	7.0	0.40	5.0	0.73	0.73	0.68
(14)SSR	16	16.0	1.00	4.0	0.78	3.0	0.36	0.38	0.33
(15)SSR	16	16.0	1.00	2.0	0.97	2.0	0.06	0.06	0.06
(16)SSR	16	16.0	1.00	5.0	0.81	5.0	0.33	0.38	0.32
(20)SSR	16	16.0	1.00	10.0	0.25	8.0	0.83	0.69	0.81
(22)SSR	16	16.0	1.00	6.0	0.72	6.0	0.46	0.25	0.43
(23)SSR	16	16.0	1.00	3.0	0.84	2.0	0.26	0.19	0.23
(26)SSR	16	16.0	1.00	5.0	0.47	4.0	0.66	0.06	0.60
(27)SSR	16	16.0	1.00	2.0	0.88	2.0	0.22	0.25	0.19
(28)SSR	16	16.0	1.00	8.0	0.53	6.0	0.66	0.63	0.63
(30)SSR	16	16.0	1.00	4.0	0.59	3.0	0.51	0.13	0.41
(31)SSR	16	16.0	1.00	11.0	0.19	12.0	0.89	0.88	0.88
(32)SSR	16	16.0	1.00	3.0	0.91	3.0	0.17	0.06	0.17
(33)SSR	16	16.0	1.00	9.0	0.50	7.0	0.69	0.44	0.66
(34)SSR	16	16.0	1.00	5.0	0.56	4.0	0.58	0.56	0.51
(35)SSR	16	16.0	1.00	6.0	0.69	4.0	0.49	0.13	0.46
(36)SSR	16	14.0	0.88	3.0	0.75	4.0	0.40	0.43	0.36
(37)SSR	16	16.0	1.00	5.0	0.59	4.0	0.58	0.44	0.53
(38)SSR	16	16.0	1.00	4.0	0.88	5.0	0.23	0.19	0.22
(39)SSR	16	16.0	1.00	12.0	0.41	9.0	0.77	0.63	0.74
(40)SSR	16	16.0	1.00	5.0	0.53	5.0	0.61	0.13	0.55
(41)SSR	16	16.0	1.00	3.0	0.88	2.0	0.22	0.13	0.19
(42)SSR	16	16.0	1.00	3.0	0.81	3.0	0.32	0.13	0.29
(44)SSR	16	16.0	1.00	3.0	0.88	3.0	0.23	0.13	0.21
(45)SSR	16	16.0	1.00	9.0	0.28	10.0	0.83	0.81	0.81
(46)SSR	16	16.0	1.00	7.0	0.56	7.0	0.61	0.44	0.57
(48)SSR	16	16.0	1.00	6.0	0.56	7.0	0.61	0.69	0.57
(49)SSR	16	16.0	1.00	6.0	0.28	10.0	0.81	0.75	0.79
(50)SSR	16	15.0	0.94	7.0	0.53	7.0	0.67	0.20	0.64
(52)SSR	16	14.0	0.88	5.0	0.64	4.0	0.53	0.21	0.48
(53)SSR	16	14.0	0.88	7.0	0.50	7.0	0.68	0.71	0.65
(57)SSR	16	15.0	0.94	7.0	0.63	7.0	0.57	0.27	0.55
(59)SSR	16	14.0	0.88	7.0	0.50	6.0	0.70	0.21	0.67
(60)SSR	16	15.0	0.94	7.0	0.33	7.0	0.79	0.80	0.77
Mean	16	15.7	0.98	6.3	0.57	6.2	0.57	0.43	0.54

**Table 5:** Genetic characteristics of 25 SSR markers analyzed in 20 *C. kanran* samples.

Marker	SS	NOBS	Availability	NG	MAF	NA	GD	Het	PIC
JCK-SSR06	20	20	1	14	0.18	16	0.92	0.90	0.91
JCK-SSR07	20	20	1	10	0.43	8	0.72	0.80	0.69
JCK-SSR11	20	20	1	15	0.18	19	0.92	0.70	0.91
JCK-SSR12	20	19	0.95	12	0.32	9	0.81	0.58	0.79
JCK-SSR13	20	20	1	10	0.30	7	0.77	0.85	0.74
JCK-SSR14	20	20	1	4	0.80	3	0.34	0.35	0.30
JCK-SSR16	20	20	1	7	0.75	6	0.42	0.45	0.40
JCK-SSR22	20	20	1	4	0.78	4	0.38	0.25	0.36
JCK-SSR26	20	20	1	7	0.40	6	0.74	0.15	0.70
JCK-SSR28	20	20	1	10	0.48	8	0.71	0.55	0.67
JCK-SSR30	20	20	1	6	0.60	5	0.56	0.30	0.50
JCK-SSR31	20	20	1	14	0.18	11	0.88	0.70	0.87
JCK-SSR33	20	20	1	12	0.33	7	0.80	0.55	0.78
JCK-SSR34	20	20	1	5	0.53	4	0.60	0.60	0.53
JCK-SSR35	20	20	1	6	0.70	6	0.48	0.10	0.45
JCK-SSR37	20	20	1	4	0.68	4	0.50	0.65	0.45
JCK-SSR39	20	20	1	14	0.33	11	0.82	0.65	0.79
JCK-SSR40	20	20	1	7	0.55	5	0.61	0.25	0.56
JCK-SSR45	20	20	1	12	0.28	9	0.82	0.75	0.79
JCK-SSR46	20	20	1	10	0.50	8	0.69	0.50	0.65
JCK-SSR48	20	20	1	11	0.53	12	0.69	0.50	0.67
JCK-SSR49	20	20	1	10	0.28	11	0.84	0.55	0.82
JCK-SSR53	20	20	1	11	0.48	9	0.73	0.65	0.71
JCK-SSR59	20	20	1	11	0.35	7	0.75	0.40	0.71
JCK-SSR60	20	20	1	8	0.45	8	0.73	0.70	0.70
Mean	20	19.96	0.998	9.36	0.45	8.12	0.69	0.54	0.66

**Table 6:** List of Cymbidium samples used for genetic diversity analysis.

Sample code	Description
JCK-01	Young sprout
JCK-02	Young sprout
JCK-03	Young sprout
JCK-04	Young sprout
JCK-05	Young sprout
JCK-06	Parent of cultured *Cymbidium Kanran* Makino
JCK-07	Purple *Cymbidium Kanran*
JCK-08	Cultivars that are being crossed with cultivated *Cymbidium Kanran* Makino
JCK-09	Agricultural Technology Institute improved variety (dark red flower)
JCK-10	Hybrid of *Cymbidium kanran* Makino and *Cymbidium goeringii*.
JCK-11	Jeokhwa (Personal name breed)
JCK-12	Jeokhwa (Personal name breed)
JCK-13	Jeokhwa (Personal name breed)
JCK-14	Jeokhwa (Personal name breed)
JCK-15	Nokhwa (Personal name breed)
JCK-16	Presumed to be Jeju *Cymbidium Kanran* Makino that spread to Japan
JCK-17	Additional sample-1 (from Wando, Korea)
JCK-18	Additional sample-2 (from China)
JCK-19	Additional sample-3 (from Japan)
JCK-20	Additional sample-4 (*Cymbidium Kanran* Makino that spread to Japan)

**Table 7:** PCR reactions and amplification conditions.

PCR reaction condition		PCR amplification condition		
Components	Vol. (μl)	Temp. (°C)	Time	Cycle No.
gDNA (10 ng/μl)	1	95	5 min	1
F^1^/R^2^ primers (10 pmol)	0.3/0.3	95	30 sec	35
2x HS^TM^ Taq Mix	5	57	30 sec	
D.W.	3.4	72	30 sec	
Final Reaction Vol.	10	72	30 min	1
