# Supplementary material for: Development of Simple Sequence Repeat (SSR) Markers from a Genome Survey of a Cymbidium kanran Makino Population in Jeju Island, Republic of Korea
Source: J Microbiol Biotechnol. 2025 Feb 24;35:e2501013. doi: 10.4014/jmb.2501.01013 (PMC11985410; doi:10.4014/jmb.2501.01013)

J. Microbiol. Biotechnol. 2025. 35: e2501013

https://doi.org/10.4014/jmb.2501.01013

Supplemantary Table and Figure

Table S1. Results of paired-end WGS using the Illumina NovaSeq6000 sequencing platform.

Sample ID	Total read bases(bp)	Total reads	GC(%)	AT(%)	Q20(%)	Q30(%)
JCK-07	73,845,942,980	480,045,980	32.99	67.01	97.38	92.55
JCK-01	75,424,996,790	499,503,290	33.17	66.83	97.22	92.34

Fig. S1. **Orchid colony zones (Native habitat protection zone, 389,879㎡) on Jeju Island, Republic of Korea**. The habitat is managed by the Orchid Exhibition Hall, located at approximately 33.2536°N latitude and 126.6016°E longitude in Seogwipo-si, Jeju, Republic of Korea.

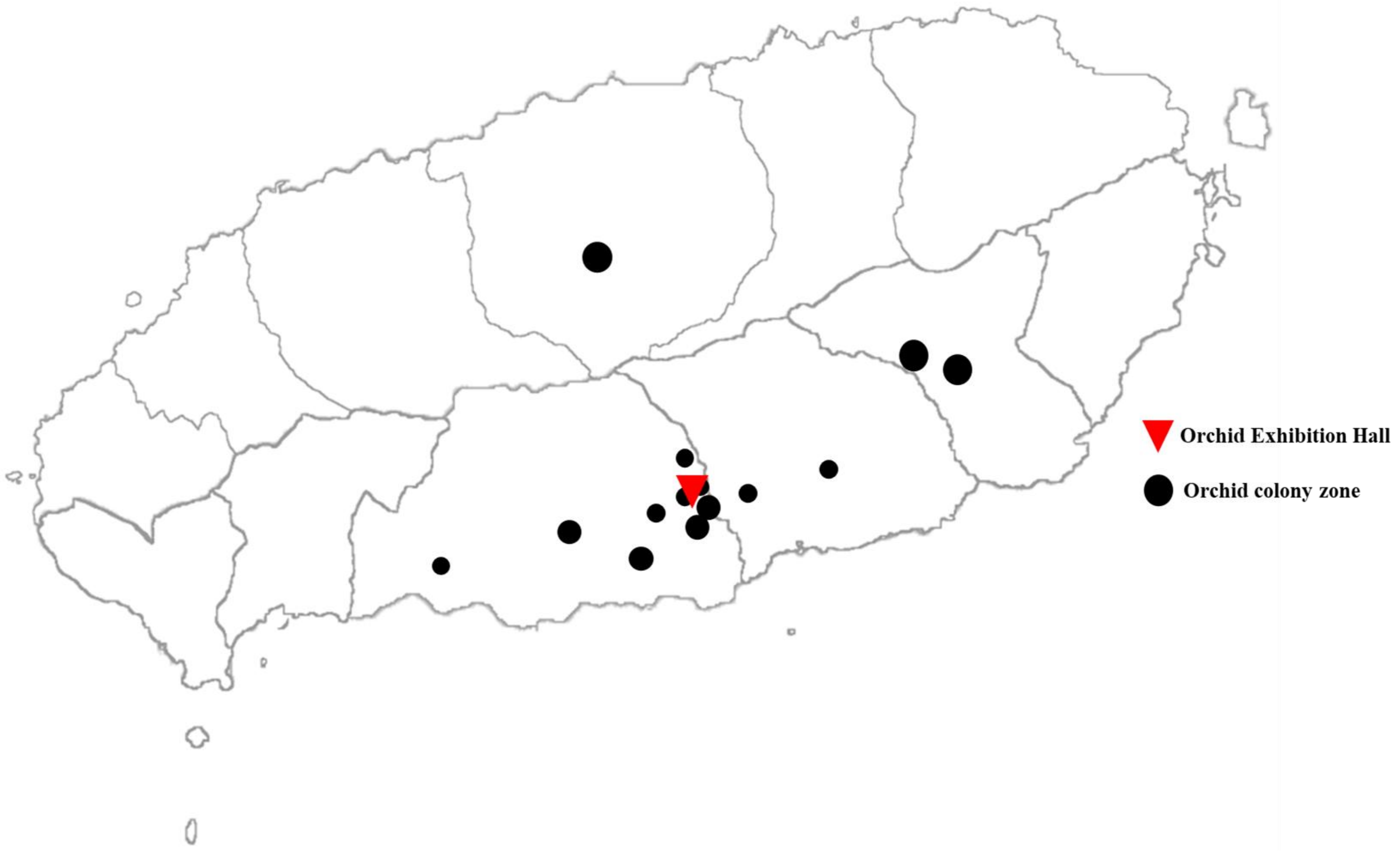

Supplement: Supplementary file 1 [file jmb-35-e2501013-supple.pdf]
